# PD-L1 expression assessment in Angiosarcoma improves with artificial intelligence support

**DOI:** 10.1016/j.jpi.2025.100447

**Published:** 2025-05-09

**Authors:** F.H. Reith, A. Jarosch, J.P. Albrecht, F. Ghoreschi, A. Flörcken, A. Dörr, S. Roohani, F.M. Schäfer, R. Öllinger, S. Märdian, K. Tielking, P. Bischoff, N. Frühauf, F. Brandes, D. Horst, C. Sers, D. Kainmüller

**Affiliations:** a Max Delbrück Center for Molecular Medicine in the Helmholtz Association; b Helmholtz Imaging, Berlin, Germany; c Charité - Universitätsmedizin Berlin, Berlin, Germany; d Humboldt-Universität Berlin, Faculty of Mathematics and Natural Sciences, Berlin, Germany; e Charité - Universitätsmedizin Berlin, corporate member of Freie Universität Berlin, Humboldt-Universität zu Berlin, Department of Pathology, Berlin, Germany; f Charité - Universitätsmedizin Berlin, corporate member of Freie Universität Berlin and Humboldt Universität zu Berlin, Department of Dermatology, Berlin, Germany; g Charité - Universitätsmedizin Berlin, corporate member of Freie Universität Berlin, Humboldt-Universität zu Berlin and Berlin Institute of Health, Department of Hematology, Oncology, and Tumor Immunology, Berlin, Germany; h German Cancer Consortium (DKTK), partner site Berlin, and German Cancer Research Center (DKFZ), Heidelberg, Germany; i Charité - Universitätsmedizin Berlin, corporate member of Freie Universität Berlin and Humboldt-Universität zu Berlin, Berlin, Germany; j Berlin Institute of Health at Charité, Universitätsmedizin Berlin, BIH Biomedical Innovation Academy, BIH Charité (Junior) Clinician Scientist Program, Berlin, Germany; k Charité - Universitätsmedizin Berlin, corporate member of Freie Universität Berlin and Humboldt Universität zu Berlin, Institute for Radiology, Berlin, Germany; l Charité - Universitätsmedizin Berlin, corporate member of Freie Universität Berlin and Humboldt Universität zu Berlin, Department of Surgery, Experimental Surgery, Berlin, Germany; m Charité - Universitätsmedizin Berlin, corporate member of Freie Universität Berlin and Humboldt Universität zu Berlin and Berlin Institute of Health, Center for Musculoskeletal Surgery, Berlin, Germany; n Inselspital, Bern University Hospital, University of Bern, Department of Medical Oncology, Bern, Switzerland; o University of Potsdam, Digital Engineering Faculty, Potsdam, Germany

**Keywords:** PD-L1 expression, Angiosarcoma, Artificial intelligence, Digital pathology, Immunotherapy, AI-assisted diagnosis

## Abstract

Tumoral PD-L1 expression is assessed to weigh immunotherapy options in the treatment of various types of cancer. To determine PD-L1 expression, each tumor cell needs to be assessed to calculate the percentage of PD-L1 positive tumor cells, called tumor proportion score (TPS). Pathologists cannot evaluate each cell individually due to time constraints and thus need to approximate TPS, which has been shown to result in low concordance rates.

Decision quality could be improved by an AI-based TPS prediction tool which serves as a “second opinion”. Establishing such a tool requires a certain amount of training data, which manifests a bottleneck for rare cancer types such as Angiosarcoma.

To address this challenge, we developed and open sourced a pipeline that leverages pre-trained and generalist models to achieve strong TPS prediction performance on limited data. Pathologists were asked to reassess patients for which their TPS strongly disagreed with the AI's prediction. In many of these cases, pathologists updated their TPS score, improving their assessment, thus demonstrating the technical feasibility and practical value of AI-based TPS scoring assistance for rare cancers.

## Highlights

• AI-assisted diagnosis improves Angiosarcoma PD-L1 tumor proportion score prediction.
• Pathologists updated their prediction after AI review and identified errors.
• Integrated generalist models in pipeline for strong performance with limited data.
• Open-sourced pipeline prediction and training code, including trained model weights.

## 1 Introduction

Angiosarcoma, a rare and aggressive cancer that arises from the endothelial cells of blood or lymphatic vessels, often has a poor prognosis.^1^, ^2^, ^3^ Treatment includes surgery, radiation, or chemotherapy, yet these modalities have shown limited improvement in patient survival.^4^, ^5^, ^6^ Immunotherapy, targeting proteins such as the programmed cell death protein (PD-1) and its ligand, PD-L1, has emerged as a promising option.^7^ PD-L1 expression, when present on cancer cells, allows cancer cells to evade the immune surveillance and worsens the prognosis.^8^, ^9^, ^10^, ^11^ PD-L1 inhibitors have shown great potential in the treatment of various cancers, including subtypes of Angiosarcoma.^12^, ^13^, ^14^

Therefore, a most accurate determination of PD-L1 expression in tumor samples is of high importance in order to identify patients who will likely benefit from PD-L1 inhibition. The current standard method to determine PD-L1 expression is the visual analysis of immunohistochemically (IHC) stained whole slide images (WSIs) by trained pathologists to estimate the PD-L1 expression tumor proportion score (TPS).^14^, ^15^, ^16^, ^17^ Pathologists have limited time to evaluate a tumor and therefore cannot assess the PD-L1 status of each cancer cell individually. Thus, approximate PD-L1 expression is performed by estimating the percentage of stained tumor tissue. Along with variabilities in staining, such an approach can lead to different PD-L1 expression assessments with a low concordance rate between pathologists.^18^, ^19^, ^20^, ^21^ These challenges underscore the benefits of a deep learning-based, quantifiable approach to accompany the pathologists' diagnosis.

Multiple deep learning approaches have been proposed for processing and analyzing immunohistochemically (IHC) stained tissue slides. For a prominent example, DeepLIIF^22^ is a framework that provides quantitative robust scoring across a variety of IHC stainings. However, DeepLIIF is tailored to nuclear staining, whereas PD-L1 is localized on the cell membrane. Motivated by a strong correlation between IHC-detected PD-L1 expression and patient response rates,^23^^,^^24^ some approaches directly target PD-L1 status prediction for lung-, head and neck- and breast cancer entities.^25^, ^26^, ^27^, ^28^, ^29^, ^30^ However, the adaptation of these approaches to Angiosarcoma PD-L1 status prediction is hindered by two factors: First, the rarity of Angiosarcoma entails that there is relatively little annotated training data available, which has been shown to be detrimental to model performance in a wide range of diverse application scenarios. Second, none of the existing PD-L1 status prediction approaches shared their code and models in a fully open source manner, thus hindering benchmarking of these approaches on new data and new cancer entities as well as dissemination to practitioners.

In response to this gap, we developed a deep learning-based approach that tackles the scarcity of training data faced in rare cancer entities. To this end our approach builds upon generalist models and the fine-tuning of pre-trained weights. Furthermore, we fully open source our approach in the form of the library *PEERCE* (PD-L1 Expression Estimation for Rare Cancer Entities).^31^ Beyond shipping code and trained models for Angiosarcoma TPS prediction, *PEERCE* allows users to train and fine-tune on their own data to improve TPS accuracy.

This open nature of our work allows for straightforward integration into standardized interfaces, such as the *EMPAIA* platform,^32^ further increasing the usage of *PEERCE* by pathologists.

Our method works by identifying tumor regions, assessing PD-L1 status at the cellular level, and aggregating these findings into a PD-L1 expression TPS. Our approach allows us to create a useful deep learning model based on limited data and annotations available for Angiosarcoma: We compare the performance of our approach to the TPS assessment of three experienced, board-certified pathologists, revealing correlation coefficients between 0.83 and 0.93. Our method, which provides a robust and interpretable cell count-based TPS, is not intended to replace the pathologist's assessment, but to complement it and provide a reliable “second opinion”. In cases of significant discrepancy between the AI-derived TPS and the pathologist's assessment, re-analysis by the pathologist can be suggested, potentially improving diagnostic accuracy.

## 2 Material and methods

### 2.1 Data

#### 2.1.1 Tissue specimens (data set)

A total of 63 patients with histopathologically confirmed angiosarcoma were included in the entire collective. The patients were presented to Charité Universitätsmedizin between 2010 and 2022. At least one representative tumor-bearing formalin-fixed paraffin-embedded tissue block was available from all 63 patients. Of these, 27 were female and 36 were male. Their age at diagnosis ranged from 27 to 96 years with an average of 66 years. Tumor sampling was performed via surgical operation for 39 patients, as well as via biopsy for the remaining 24 patients (see Table 1). The study was approved by the local ethic committee (approval number EA4/012/22) and was performed in accordance with the Declaration of Helsinki.

**Table 1:** Characteristics of angiosarcoma patients.

Category	Subcategory	Value
Total Patients	Angiosarcoma	63
Sex Assigned at Birth	Male	36
	Female	27
Sampling Method	Surgical Operation	39
	Biopsy	24
Age at Diagnosis	Average	66
	Range	27–96
PD-L1 TPS	<1 %	48
	1–50 %	9
	≥50 %	6
	Range	0–100 %

Following H&E staining and additional immunohistochemistry, all slides were annotated with regions of interest (ROIs) indicating tumor presence. In addition to the 63 angiosarcoma patients, 16 sarcoma cases were available that were initially incorrectly classified as angiosarcoma during routine diagnostics, however this diagnosis was not confirmed during the course of the study. After digitization, 27,916 patches of confirmed angiosarcoma were available and 6101 patches of sarcomas that could not be clearly classified as angiosarcoma. In total, 34,017 patches were extracted for training. During cross-validation, only confirmed angiosarcoma patches were used in the validation set.

Due to the nature of staining procedures on adjacent tissue slices, perfect cell alignment between H&E and PD-L1 slides is not always possible. In 61 out of 79H&E tissue samples, H&E and PD-L1 slides were so well-aligned that tissue structures closely overlap in both slides. Out of these well-aligned slides, 48 WSIs with 20,739 patches came from 47 patients with verified angiosarcoma. The remaining 14 WSIs, each from a unique patient, contributed 4810 patches. These were also used for training but not for validation.

#### 2.1.2 H&E and PD-L1 staining

After formalin fixation and paraffin embedding (FFPE), 3 μm tissue sections were cut, afterwards deparaffinized by heat (70 °C), H&*E*-stained and covered by Sakura Tissue-Tek Prisma® Plus.^33^ PD-L1 staining was performed using DAB-staining on a Leica Bond.^34^ The PD-L1 antibody was used at 1:200 dilution (Cell Signaling Technology Inc., clone E1L3NR, catalog number 13684S). After staining, tissues were rehydrated in graded alcohols and xylene. Finally, all slides were film coverslipped using Sakura Tissue-Tek Film®.^33^

#### 2.1.3 Digitization

All WSIs were scanned via a 3DHISTECH PANNORAMIC 1000 scanner^35^ at 40× magnification, yielding a resolution of 0.25 μm per pixel. At this magnification, WSI dimensions ranged widely from *15840 × 22496* pixels to *184992 × 91136* pixels. The patches, to be utilized by the convolutional neural network (CNN), were extracted from the WSI at 20× magnification. At this downscaled magnification, WSIs ranged from *7920 × 11248* pixels to *92496 × 45568* pixels. Patches, as well as the WSIs themselves, were retrieved in RGB format.

#### 2.1.4 Annotation

For annotation purposes Omero Plus^36^ was used. In the process of creating the dataset used for training and evaluation, annotations were conducted at two distinct levels, the tumor area annotation and the cell type annotation.

##### 2.1.4.1 Tumor area annotation

On the WSI level, tumor ROIs were annotated to mark all tumor regions within the WSI. A pathologist, specialized in sarcoma pathology, recognized, and marked tumor regions. All WSIs have tumor areas which were annotated. Most WSIs just have one tumor ROI, while others possess up to 23 tumor ROIs, with an average of 4.83 tumor ROIs per WSI.

##### 2.1.4.2 Cell type annotation

**Fig. 1:**
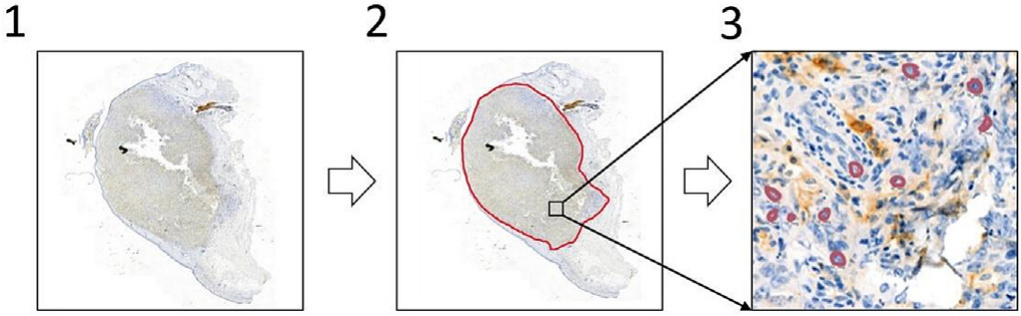
Annotation process. The slide is analyzed (1), and tumor regions of interest (ROIs) are annotated WSI-wide (2). A small number of 512 × 512 patches were then taken from each investigated WSI to individually annotate the cell- and staining type (TC+, TC- or OC) in a subset of the patch's cells (3).

The study involved detailed cell type annotation on 512 × 512 patches without specifying cellular structures. While some PD-L1 expression scores focus on both tumor and immune cell PD-L1 expression or immune cell scoring alone, we focused exclusively on tumor cell PD-L1 expression, TPS, for this study, as TPS represents a widely validated biomarker for immunotherapy patient selection.^37^^,^^38^ Annotation of immune cells was therefore not required. Cells were categorized into PD-L1 positive tumor cells (TC+), PD-L1 negative tumor cells (TC-) and non-tumor (other) cells (OC). OCs include, among others, lymphocytes, plasma cells, neutrophils and macrophages, although their lineage differentiation was not investigated further. Marking cells without specifying their exact outline sped up annotations. The process is visualized in Fig. 1.

Patches for annotation were chosen using two methods: directly from tumor ROIs to ensure tumor cell presence, and through a CNN that detects tumor patches in WSIs. This CNN is further described in the Tumor Patch Detection section and may in rare cases by mistake identify non-tumor areas. These CNN-identified patches were included in the training set to mimic the variety in WSI-wide predictions. From 65 WSIs of 63 verified angiosarcoma patients, 451 patches were annotated by a pathologist with 7487 cells including 2413 OCs and 5074 tumor cells (708 TC+ and 4366 TC-). Additionally, 13 WSIs from 13 patients provided 57 patches with 1072 cells (453 OCs, 619 tumor cells; 132 TC+ and 487 TC-) for training only, not validation, as their tumor type was unclassified. This amounted to 8559 annotated cells across 76 patients in total, consisting of 840 PD-L1 TC+ cells, 4853 PD-L1 TC- cells, and 2866 OCs.

##### 2.1.4.3 Assessment of the PD-L1-expression tumor proportion scores

The TPS indicates the ratio of PD-L1 positively stained tumor cells in relation to all tumor cells. It is given as a percentage:

$${\mathrm{ExpressionScore}}_{\mathrm{PD}-L1}=\frac{{\mathrm{TC}}_{+}}{{\mathrm{TC}}_{+}+{\mathrm{TC}}_{-}}$$

where ${\mathrm{TC}}_{+}$ is the number of positively PD-L1 stained tumor cells, whereas ${\mathrm{TC}}_{-}$ represents the number of negatively PD-L1 stained tumor cells.

Three pathologists independently assessed PD-L1 expression on all available angiosarcoma WSIs, resulting in three different TPS for each slide. We chose a discrepancy from the AI's prediction of more than 10 percentage points to prompt the respective pathologists to re-evaluate their TPS assessment, and potentially revise the score. Only the initial TP scores were used to evaluate the predictive performance of the deep learning model.

### 2.2 Data preprocessing

Data Preprocessing steps include tissue patch extraction via Otsu thresholding^39^ as well as a series of patch augmentations to improve model generalizability, as detailed in the following.

#### 2.2.1 Tissue patch identification and extraction

The WSIs provided often comprise sizes larger than 10,000 × 10,000 pixels, being too large to be processed by a CNN in one pass. Hence, we split the WSI into smaller patches for subsequent processing, as is usually done when applying deep learning techniques to WSIs.^40^^,^^41^

The WSIs also harbor white spaces (tissue cutting artifacts and tissue structure features). As only tissue patches are needed for further processing and in order to save computational resources, we used Otsu thresholding^39^ to distinguish tissue regions from the white background. We downscaled the WSI by a factor of 16 and slightly modified it to improve tissue detection performance. There are some WSIs with black edges around the slide, resulting in problematic thresholds. We excluded these by setting all pixels, whose channel values were lower than 20 for all color channels, to white. For PD-L1 WSIs, downscaled WSI was converted into grayscale. For H&E WSIs we converted the downscaled WSI to Haematoxylin-Eosin-DAB (HED) color space, using the eosine channel alone for Otsu thresholding. In rare cases, Otsu's method yielded high thresholds which exclude tissue. To avoid tissue exclusions, we established a fixed upper-bound threshold.

Applying the identified thresholds onto the downscaled WSI image yielded our tissue mask. Using the tissue mask, we iterated through all potential patch locations. As the tissue mask was downscaled, we used a step size of 32 pixels, both vertically and horizontally. Each 32 × 32 pixel patch, corresponding to a 512 × 512 pixel patch within the overall WSI, was then individually evaluated.

The 32 × 32 pixel patch was divided into 16 smaller, equally sized 8 × 8 pixel sub-patches, on which the number of thresholded tissue pixels was counted. If five or more pixels of the sub-patch were considered tissue pixels, it was considered to be a tissue sub-patch. Patch tissue coverage was then calculated as the number of sub-patches covered in tissue divided by all sub-patches. If this coverage was more than 60 %, the entire patch was considered a tissue patch. For such patch locations, the coordinates were converted back to the corresponding 512 × 512 pixel coordinates of the original WSI and stored in a list of suitable tissue patch locations.

When we extracted patches from both PD-L1 and the aligned H&E, we calculated an overlapping tissue mask by performing a logical ‘and’ operation on both tissue masks. In some cases, tissue was missing from one of the two WSIs, even though they are well aligned. Using an overlapping tissue mask ensured that only tissue-containing patches for both, PD-L1 and H&E, were extracted.

The number of identified tissue patches generally ranged from around 50 to more than 6000, per WSI, with a mean of 1846. For training purposes, 500 tissue patches were extracted from WSIs with more than 500 tissue patches, while all patches were extracted for WSIs with less than 500 patches identified. We also created a tumor mask for each patch based on provided Omero annotations.

#### 2.2.2 Patch augmentation

During the training process, data augmentation techniques were used to enhance performance and generalizability of our model. For our pipeline, we employed the Albumentations library,^42^ an efficient and adaptable tool, which allows the user to apply identical augmentation operations to more than one image. This was useful in cases where we fed both, H&E and PD-L1 patches, into the CNN.

First, we employed horizontal and vertical flipping with a 50 % probability for each flip. Then, various affine transformations were applied, including random translation, scaling and shearing, all within a specified range. We also included color jittering, which randomly alters brightness, saturation, contrast and hue, all again within a specified range.

As the model weights were pre-trained on ImageNet, we normalized our patches to resemble the mean and standard deviation of that dataset. Normalization was applied to all patches, including those used for validation, while all other augmentations were only applied during training.

### 2.3 Artificial intelligence

**Fig. 2:**
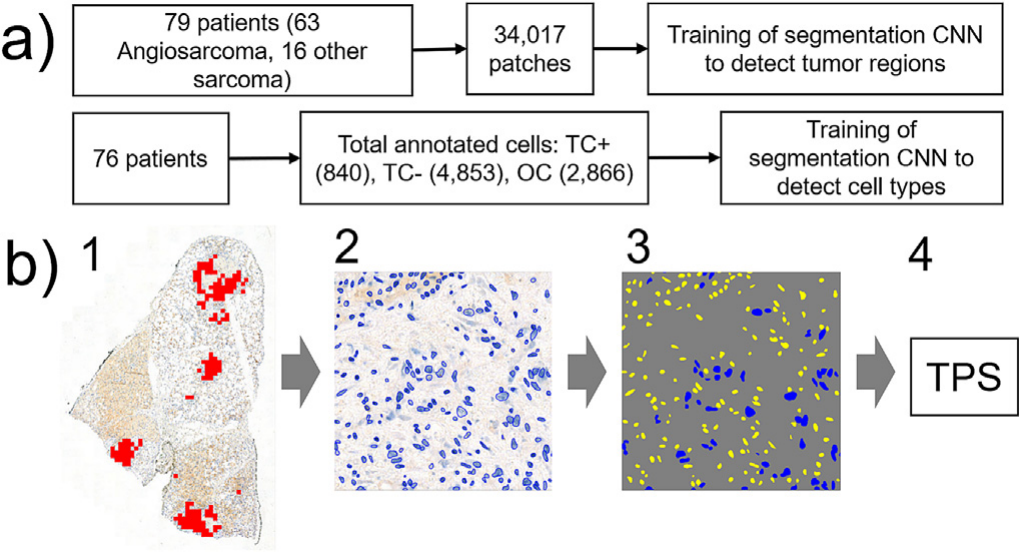
CNN Training process. a) Shown is the number of patients and patches utilized to train the segmentation CNN to detect tumor regions, as well as the number of patients and annotated cells employed to train for the segmentation of cell types. b) Visualization of the deep learning pipeline: First, all 512 × 512 patches containing tumor regions are identified via a U-Net CNN (1). After this, cell instances in the identified patches are determined via Cellpose (2). Then, a U-Net predicts the cell type for each cell instance (3). The blue colored cells are TC-, while the yellow ones are OC. Finally, cell type counts are aggregated over all patches identified in (1). The tumor prediction score (4) is then calculated via division of the number of PD-L1 positive tumor cells by all tumor cells identified within the whole-slide image. (For interpretation of the references to color in this figure legend, the reader is referred to the web version of this article.)

Our deep learning approach for PD-L1 expression TPS estimation follows a three-step pipeline (Fig. 2): First, it identifies tumor patches. Then it detects and classifies cells within these patches into PD-L1 expressing tumor cells (TC+), non-expressing tumor cells (TC-), and other cells (OC). Finally, our approach aggregates the identified cell types among all WSI tumor patches to calculate the WSI wide PD-L1 expression TPS. All methods were evaluated via five-fold cross-validation, with 80 % of the data used for training and 20 % for testing in each fold.

#### 2.3.1 Tumor patch detection

To detect whether a patch consists of tumor tissue or not, we used a U-Net,^43^ an encoder-decoder neural network architecture. We chose the EfficientNetV2 M architecture^44^ as our encoder, with weights pretrained on ImageNet.^45^ We used the standard decoder with randomly initialized weights. In cases where we fed both, H&E and PD-L1 patches, into the model, we increased the number of input channels of the model from three to six.

We used PyTorch^46^ as our core deep learning library. The U-Net architecture implementation was sourced from the Segmentation Models Pytorch library,^47^ while the EfficientNet encoder model was from Pytorch Image Models.^48^ The U-Net is trained for 50 epochs, a number after which our tests didn't show any further improvements. Optimization was carried out via the AdamW^49^ optimizer with weight decay to avoid overfitting. The learning rate starts at 0.001 and is continuously decreased by a cosine annealing learning rate scheduler to optimize more fine-grained in later epochs. The chosen batch size was 8 and the model was trained using a cross-entropy loss function.

For each patch it receives as input, the U-Net outputs a 512 × 512 segmentation mask with two channels. One channel predicts the non-tumor likelihood, while the other predicts tumor likelihood for each pixel. Softmax was applied for each pixel individually, resulting in a channel where tumor likelihood for each pixel was scored in a range between zero and one.

To create an overall tumor score, we calculated the average over all the pixel values of the tumor likelihood channel. This way, we obtained an overall tumor score between zero and one for each patch. To reduce the occurrence of false positives, we used a threshold of 0.6 to determine relevant tumor patches. This threshold was determined through validation testing to minimize false positives and maximize recall. In some cases, the number of identified tumor patches within a WSI was smaller than 10. In these cases, we lowered the tumor detection threshold so that there were at least 10 identified tumor patches.

For evaluation, precision and recall were used to assess the effectiveness of our U-Net model to identify tumor patches across the WSI. In addition, Dice coefficient was used to examine prediction performance at the pixel level, by comparing the overlap between the model's prediction and manual annotations. All evaluation metrics for tumor patch detection are the result of a five-fold cross-validation with three independent runs for each fold.

#### 2.3.2 Tumor cell and staining detection

To detect tumor cells and their staining, cell outlines were identified using Cellpose,^50^ a deep learning framework capable of segmenting cell nuclei instances. To overcome detection challenges due to PD-L1 staining, an additional transform using the DeepLIIF^22^ framework was applied. Following this, a U-Net model was trained to classify cells into different types, such as tumor-cell positive, tumor-cell negative or other non-tumor cells. The counts of identified cell types were aggregated for the whole WSI and lead to the calculation of the PD-L1 expression score.

##### 2.3.2.1 Cell outline detection via Cellpose

For cell outline detection, we utilized Cellpose,^50^ a deep learning-based cell segmentation model capable of accurately segmenting cell nuclei from microscopic images. Cellpose generalizes well without the need to retrain or fine-tune the model, making it ideally suited for use in our pipeline.

Before feeding a patch into the model, we converted the patch into grayscale and inverted it. Inverting allows the patch to more closely resemble the data on which Cellpose was trained. For model prediction, we employed nuclei detection with the nuclei diameter being set to 15 pixels. We enabled network averaging, where four built-in CNNs make predictions that are averaged, as well as augmentation. Such a prediction is non-deterministic but has been shown to improve cell detection performance. All other parameters are left at their default value.

In some PD-L1 stained cases, the patch contained a significant amount of brown immunohistochemical staining. This proved challenging, as brown color is similar to the blueish nucleus color in a grayscale image, making it difficult for Cellpose to correctly detect cell nucleus contours. To overcome this issue, we utilized a sub-model from the DeepLIIF framework,^51^ which consists of a ResNet^52^ based generator that transforms any immunohistochemistry image into a hema-only slide. We then compared the number of identified cell ROIs, first for the original PD-L1 patch and second for the hema-transformed patch, selecting the Cellpose outlines from the patch with the most detected cells. In case of a tie, we use the outlines of the original patch.

##### 2.3.2.2 U-net cell type segmentation

Using U-Net segmentation, we first classify each pixel into one of four classes:

• Tumor-cell positive (TC+) - a tumor cell with PD-L1 expression,
• Tumor-cell negative (TC-) - a tumor cell without PD-L1 expression,
• Other non-tumor cell type (OC) - all other cells, and
• Background

We used an EfficientNet B5 encoder backbone^53^ for our U-Net model, pretrained on the ImageNet^45^ dataset. This consistent choice of architecture and pre-training helped to ensure uniformity across different segmentation tasks within our pipeline, as well as straightforward reproducibility.

The model was trained for 25 epochs with AdamW^49^ optimization. The learning rate starts at 0.001 and decreases via a Cosine Annealing.^49^ As for tumor patch detection, we used the cross-entropy loss function, a standard choice for multi-class classification or segmentation problems, which allowed us to mask unannotated pixels.

The number of annotated cells was only a small fraction, approximately 20 to 30 cells of the several hundred cells found in most of the identified tumor patches. Due to the masking-out of most non-annotated cell pixels, the background class has significantly more pixels compared to the cell type classes, resulting in a class imbalance. To address this, we decreased the weight of background pixels in the loss function to 0.0004, while other classes (TC+, TC- and OC) are assigned a weight of 1. This way, the loss of the model is less biased towards the background.

Finally, softmax was applied to each pixel, transforming the raw output of the model into a probability distribution across the four classes. This enabled the identification of cell type and staining for each pixel which later-on allowed us to determine the cell type and staining for each cell outline.

##### 2.3.2.3 Identification of cell type from predicted pixels

The U-Net creates a segmentation mask with softmax predictions for each pixel. Combined with the cell outlines obtained from Cellpose, we can leverage these pixel-level predictions. To do this, we created a mask for each cell outline and overlaid it on top of the U-Net's softmax prediction mask. The softmax scores of all pixels within the cell mask were then averaged, yielding a single softmax score vector for each cell outline.

This vector represents the aggregated prediction of each class (TC+, TC-, OC and background) for the whole cell within the cell outline. We did not consider the background class in our cell type prediction as the outline only included cellular elements. Using the remaining classes, the cell type was then determined via the highest softmax score. We evaluated cell type prediction performance via five-fold cross-validation with three independent runs for each fold.

#### 2.3.3 Calculation of WSI PD-L1 tumor proportion score

After the identification of cell type composition within each identified WSI tumor patch, we then aggregated this data to calculate the WSI-wide PD-L1 expression score. To compute this score, we counted the number of TC+ and TC- cells across all identified tumor patches within the WSI. The PD-L1 TPS score is then determined via the division of TC+ cells by all tumor cells.

During cross-validation, the AI predicted PD-L1 TPS scores for all 63 verified angiosarcoma cases, with each angiosarcoma case being in the model's validation set for one fold. The results were then compared to the evaluations made by three pathologists, who independently assessed each case. These experts assessed membrane-bound PD-L1 expression using established criteria. The key validation metric was the correlation between the pathologists' assessment and the AI-generated scores.

## 3 Results

### 3.1 Tumor patch detection

**Fig. 3:**
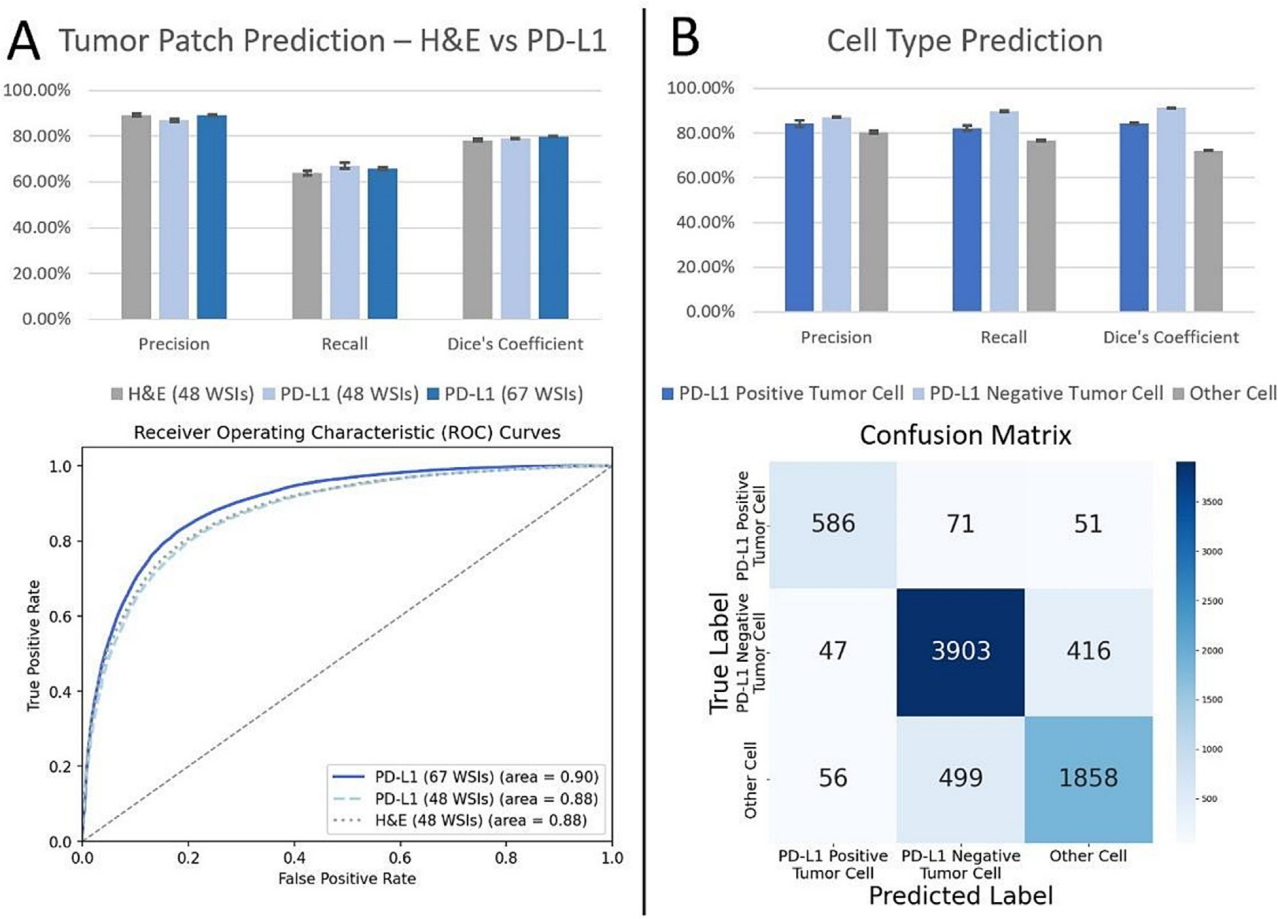
Tumor patch and cell type prediction accuracies. (A) Comparison of H&E and PD-L1 staining for precision, recall and Dice coefficient. The ROC curve further compares both methods indicating slightly better performance of PD-L1 staining. (B) Cell type classification performance through precision, recall and Dice coefficient visualized via bar chart and confusion matrix. The Dice coefficient is calculated through the actual semantic segmentation overlap. Error bars represent the standard deviations across three experimental runs.

Comparing the tumor patch detection performance of PD-L1 and H&E staining, our analysis revealed that both HE staining and PD-L1 immunohistochemistry performed well, but with subtle differences. As shown in Fig. 3, the H&E model achieved a slightly higher precision of 89.17 % (SD 0.75), compared to the PD-L1 model's precision of 87.01 % (SD of 0.60). The PD-L1 model was superior at both recall and at the Dice coefficient. For recall, the PD-L1 model achieved 67.07 % (SD 1.29) compared to the H&E model's performance of 63.85 % (SD 1.01). For the Dice coefficient, the PD-L1 model achieved 0.790 (SD 0.001), while the H&E model achieved 0.783 (SD 0.003).

Given the similar efficacy of both modalities, we decided to use only PD-L1 slides for our subsequent analyses, allowing us to utilize a more extensive dataset, with a total of 67 WSIs from 63 distinct patients. Within this larger dataset, we managed to not only improve the Dice coefficient, but also to find a better balance between precision and recall. Specifically, the model achieved a precision of 89.19 % (SD 0.45). In terms of recall, the model had a mean score of 65.76 % (SD 0.76). The Dice coefficient reached a mean value of 0.799 (SD 0.002), indicating a consistently high level of performance.

### 3.2 Cell type prediction

In our analysis for cell type prediction, we assessed all three cell types individually, TC+, TC- and OC. We excluded the background class from validation and calculated the Dice coefficient by aggregating the Dice scores over all annotated cell masks.

Cell type prediction results are illustrated in Fig. 3. For TC+ cells, the model achieved a mean Dice coefficient of 84.45 % (SD of 0.367). The precision was at 84.30 % (SD 1.475), and the recall was 82.16 % (SD 1.072). For TC- cells, the performance was significantly better, with mean Dice coefficient 91.27 % (SD 0.044), precision 87.13 % (SD 0.088), and the recall 89.70 % (SD 0.253). In the case of OC, the mean Dice coefficient was 72.18 % (SD 0.148), the precision was 80.46 % (SD 0.667), and the recall was at 76.75 % (SD 0.412).

These results indicate that the model performs best for TC- cells, followed by TC+, and then OC. OCs are typically smaller than cancer cells and were therefore pixel-wise underrepresented in the dataset. The overall cell type prediction accuracy for all three cell classes combined is 84.81 % (SD 0.057).

### 3.3 TPS prediction

**Fig. 4:**
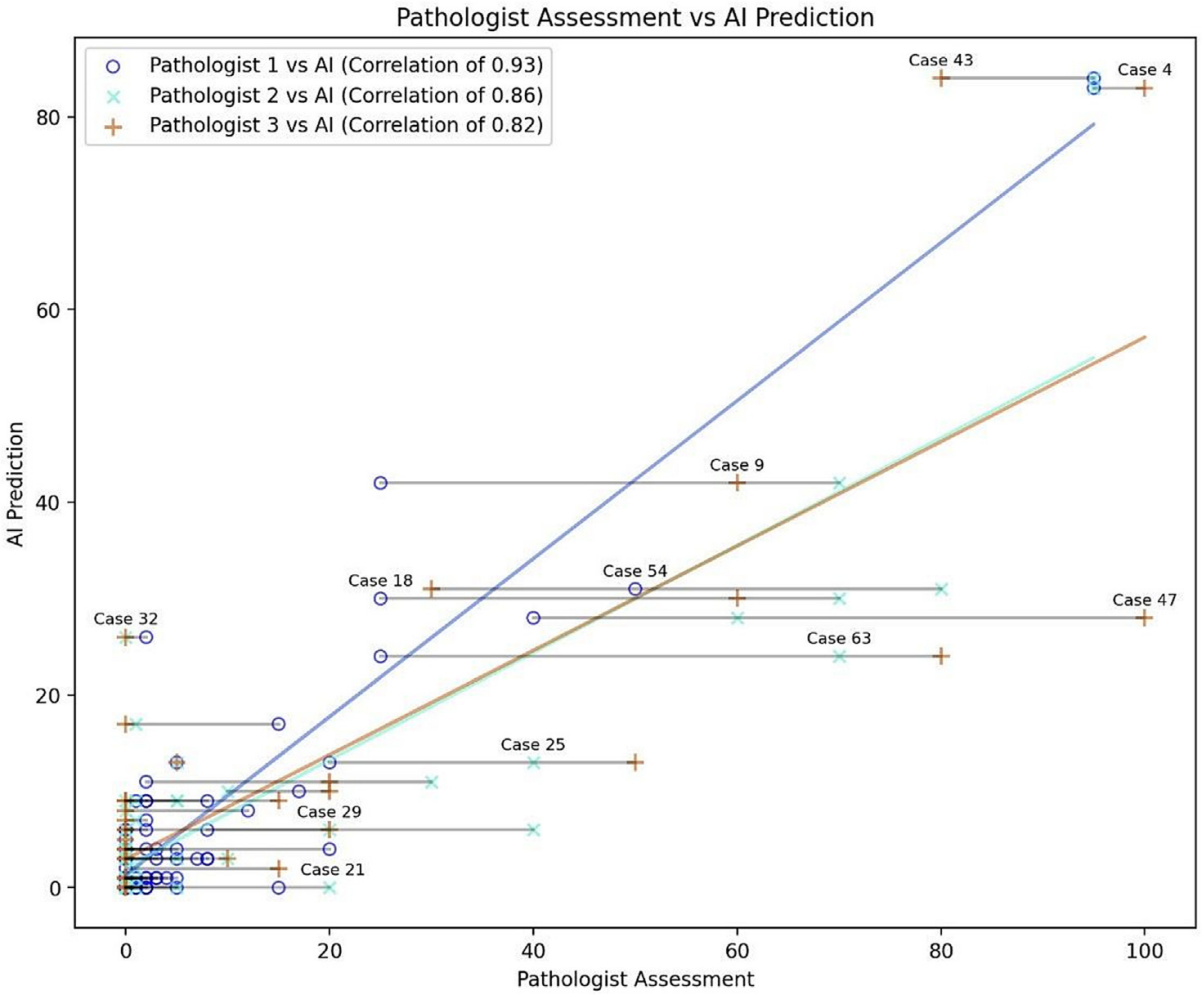
Scatterplot showing PD-L1 TPS assessment of the three pathologists, as well as the AI's prediction. Gray lines connect the individual PD-L1 expression predictions for each WSI.

Comparing the PD-L1 TPS predictions of our AI system with the independent assessment of three pathologists revealed an average correlation coefficient of 0.87. The individual correlations were 0.82, 0.86 and 0.93. The pathologists' assessment also deviated substantially for various cases as illustrated in Fig. 4.

Four patients had multiple slides. In these cases, the TPS scores across slides for the same subject were very similar, thereby emphasizing the robustness of our approach. In one case, where all pathologists predicted a TPS of 0 %, our pipeline predicted a TPS of 0.08 % for one slide and 0.20 % for another. In another case, the pathologists predicted an average TPS of 17.33 % with 2 %, 30 % and 20 % individually. The TPS predictions of our AI were 12.33 % and 11.39 %. For another patient with a pathologist assessment of 0 %, 1 %, and 15 %, our model predicted 0.35 % and 2.15 % for the individual slides. In one case, the TPS predictions were at 0.03 % and 9.04 %. Closer examination revealed that this case had one slide from the primary tumor while the other slide was derived from a liver metastasis, which could account for the difference in TPS scores.

To make our model more applicable for clinical decision-making, we introduced a cut-off TPS score of 20 %. With this cut-off, the model's accuracy rate reached an average of 95.24 % with individual pathologist accuracies of 96.83 %, 93.65 % and 98.41 %. Specifically, only 2, 4, or 1 case were differentially assessed, respectively.

### 3.4 Pathologist reassessment

To evaluate our model's performance on whole slide PD-L1 expression prediction, three pathologists independently assessed PD-L1 expression TPS in angiosarcoma using established histopathological criteria. Tumor cells with membrane-bound PD-L1 expression were evaluated in relation to all tumor cells and a respective score was determined. Comparing the pathologists' evaluation and our AI model, we chose a discrepancy of more than 10 percentage points as an indicator of potential immunohistochemical misscoring. In these cases, the pathologist's carried out a re-evaluation.

In the course of this process 17 of the 63 patients were flagged for a review by at least one pathologist, resulting in 33 of all 189 assessments being re-evaluated (17.46 %). The pathologists changed their PD-L1 expression estimate in 27 cases (17 patients), not changing their score in the 6 remaining cases.

**Fig. 5:**
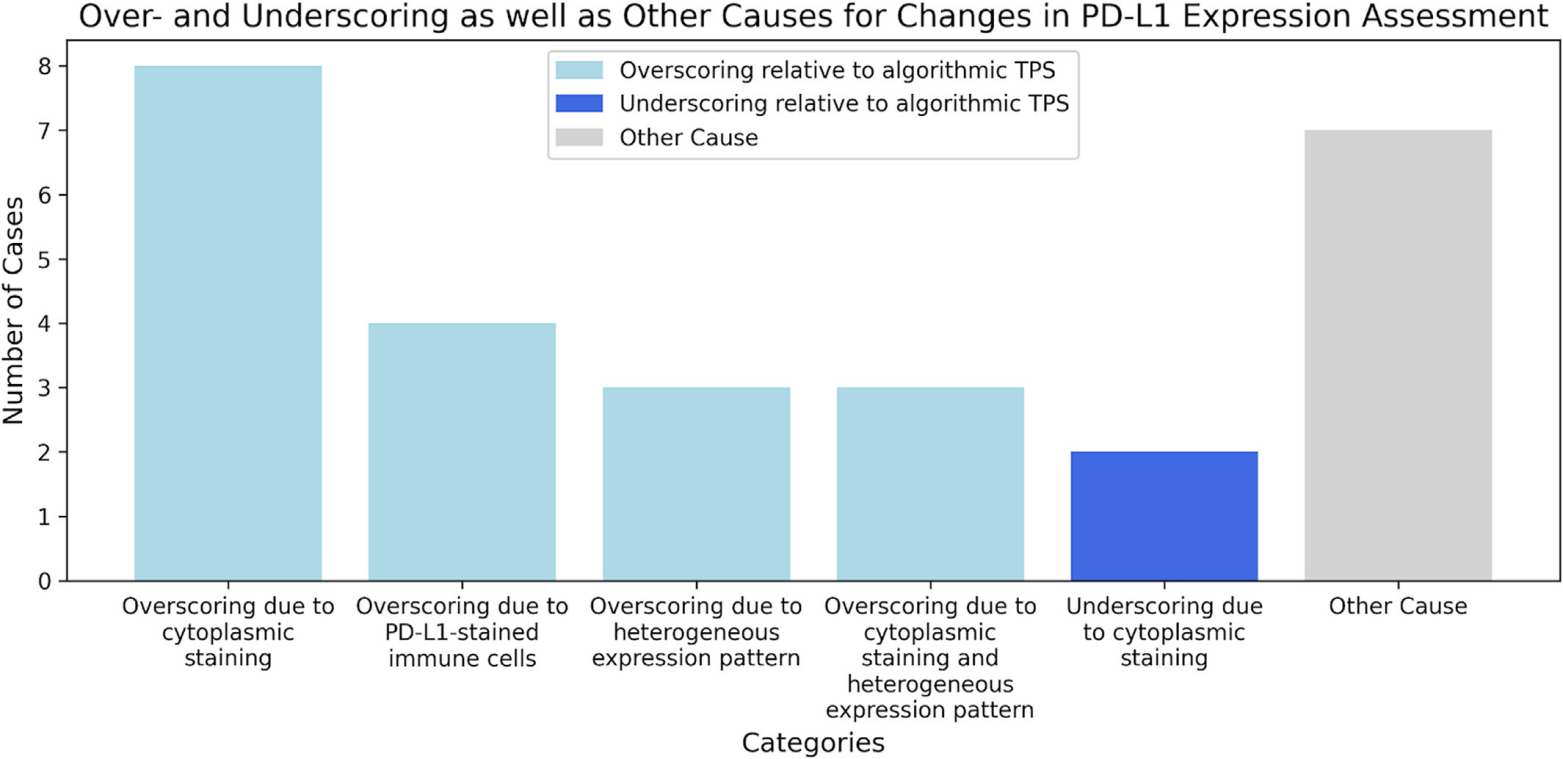
Distribution of over- and underscoring, as well as other causes leading the pathologists to update their PD-L1 assessment. These self-reported errors were identified in a secondary review process, where discrepancies between AI and pathologist prediction prompted a renewed assessment.

When pathologists revised their assessment, they furthermore provided their rationale for doing so. These reasons are shown via a bar chart in Fig. 5. It was found, for example, that cytoplasmic staining in tumor cells was overscored in 11 instances, and underscored in 2. Other diagnostic challenges were heterogeneous expression patterns, as well as PD-L1-stained immune cells.

## 4 Discussion

In our study, we developed a data-driven deep learning pipeline for biomarker prediction in small tissue cohorts with limited data availability, focusing on PD-L1 expression in angiosarcoma.

The diagnostic inaccuracy in everyday's medical practice leads to misinterpretation and therefore to overscoring of strong PD-L1 expression and underscoring of weak PD-L1 expression in angiosarcoma. We found that receiving feedback from our AI-based tool, *PEERCE*, allows pathologists to identify errors and potentially improve their scores, which may lead to improvements in immunotherapy treatment and ultimately, patient survival.

**Fig. 6:**
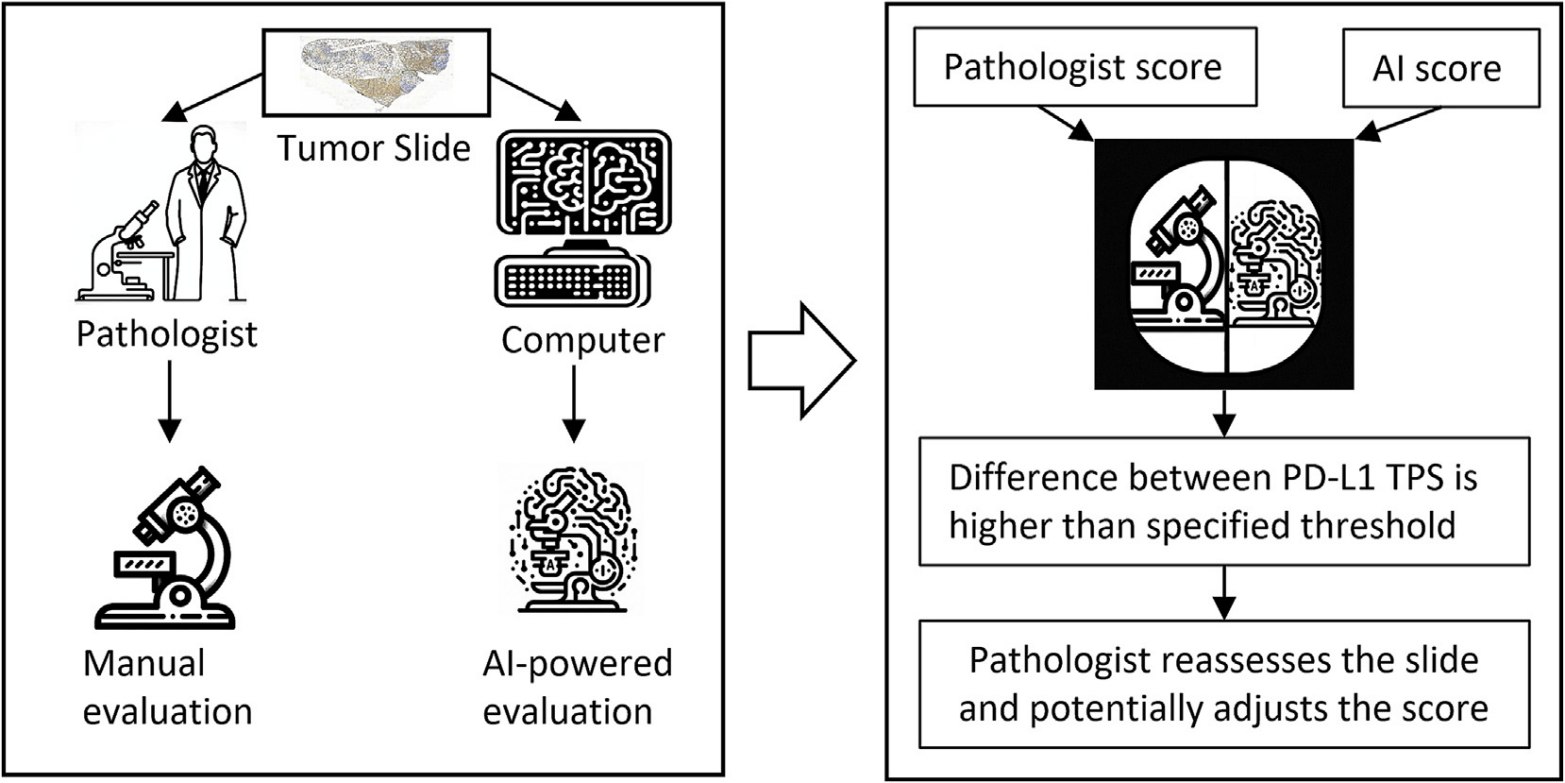
PD-L1 Expression Evaluation Workflow. An AI-based method and a pathologist independently determine PD-L1 expression on a tumor slide, resulting in TC scores. Significant TC score variances trigger a detailed pathologist reassessment. The extended evaluation time enables score correction, illustrating how AI facilitates improved accuracy in time-sensitive diagnostic tasks.

Our findings align with related work in demonstrating the value of automated image analysis for PD-L1 assessment,^25^, ^26^, ^27^^,^^29^^,^^30^ where recent work also showed that AI assistance can significantly improve concordance between pathologists in PD-L1 scoring.^28^ In line with the underlying goal to standardize PD-L1 assessment in other cancers, our tool strives to provide reliable quantitative scoring, thereby improving accuracy for angiosarcoma. For rare cancers, AI assistance as a “second opinion” has a particular value, as pathologists may have limited experience with PD-L1 scoring compared to more common malignancies. Our findings demonstrate that AI can help identify potential misinterpretations, particularly in cases with challenging features such as heterogeneous expression patterns or the presence of PD-L1 positive immune cells. Thereby, our tool provides pathologists with a quantitative backup for their assessment decisions. The proposed workflow is depicted in Fig. 6.

Our work showcases the feasibility and impact of AI-based assistance in PD-L1 expression assessment for the case of angiosarcoma as a rare cancer entity. By making *PEERCE* available to the wider research- and clinical community under Massachusetts Institute of Technology license, we aim to empower the creation of advanced diagnostic tools for further cancer types, particularly rare ones such as soft tissue sarcomas^54^^,^^55^ and especially for entities like liposarcomas^56^ and osteosarcomas.^57^

Our study is not without limitations. The inherent scarcity of data faced for rare cancer entities such as angiosarcoma poses a challenge in creating an external test set, limiting our ability to validate the generalizability of our findings. Furthermore, a significant challenge encountered was the differentiation between tumor cells and other nonmalignant cells. This is particularly intricate in angiosarcoma, which can arise anywhere in the body, resulting in extensive variability in the appearance and characteristics of the nonmalignant tissue cells in the tumor microenvironment. Moreover we used the E1L3N PD-L1 antibody clone which, while having shown high concordance with FDA-approved clones,^58^ is not FDA-approved as a companion diagnostic. Finally, as mentioned above, we employed a cut-off at 20 % for our evaluation. There are currently no established PD-L1 expression cut-offs for treatment response in angiosarcoma. Current ESMO guidelines report that PD-L1 inhibitors can be considered in treatment^59^ with case reports showing remission in scalp angiosarcomas,^60^ but specific thresholds remain to be established. Therefore, we used a threshold of 20 % as is used in other tumor entities.^61^

Looking forward, there are several avenues for enhancement: incorporating further foundation models, in particular very recent ones that specifically target H&E,^62^, ^63^, ^64^ could further reduce the need for expert annotations and further refine the model's predictive capabilities.

In summary, our study demonstrates the technical feasibility and practical value of advanced machine learning models in a medical context with limited data. These models not only augment human expertise but also open up new possibilities for future research and enhancements in the field of medical diagnostics.

## Funding

This work was supported by the 10.13039/100005930German Research Foundation RTG 2424, 10.13039/501100009318Max-Delbrueck-Center for Molecular Medicine in the Helmholtz Association (MDC) and Helmholtz Imaging.

## Declaration of competing interest

The authors declare that they have no known competing financial interests or personal relationships that could have appeared to influence the work reported in this paper.
